# Intestinal epithelial PTPN2 limits pathobiont colonization by immune-directed antimicrobial responses

**DOI:** 10.1080/19490976.2025.2559029

**Published:** 2025-09-15

**Authors:** Pritha Chatterjee, Marianne R. Spalinger, Charly Acevedo, Alina N. Santos, Casey M. Gries, Salomon M. Manz, Vinicius Canale, Ali Shawki, Anica Sayoc-Becerra, Hillmin Lei, Meli’sa S. Crawford, Lars Eckmann, James Borneman, Declan F. McCole

**Affiliations:** a Division of Biomedical Sciences, University of California, Riverside, Riverside, CA, USA; b Department of Gastroenterology and Hepatology, University Hospital Zurich, University of Zurich, Zurich, Switzerland; c Division of Gastroenterology, University of California, San Diego, La Jolla, CA, USA; d Department of Microbiology and Plant Pathology, University of California, Riverside, Riverside, CA, USA

**Keywords:** Adherent-invasive E. coli (AIEC), Antimicrobial Peptide, Barrier Function, Interleukin-22, Intestinal Permeability, Pathobiont, Tight Junctions

## Abstract

Loss of activity of the inflammatory bowel disease (IBD) susceptibility gene, protein tyrosine phosphatase non-receptor type 2 (*PTPN2*), is associated with altered microbiome composition in both human subjects and mice. Furthermore, expansion of the bacterial pathobiont, adherent-invasive *E. coli* (AIEC), is strongly linked to IBD pathogenesis. The mechanism by which intestinal epithelial cells (IEC) maintain equilibrium between commensal microbiota and immune cells to restrict invading pathobionts is poorly understood. Here, we investigated the role of IEC-specific PTPN2 in regulating AIEC colonization. Tamoxifen-inducible, intestinal epithelial cell-specific *Ptpn2* knockout mice (*Ptpn2*^∆IEC^) and control *Ptpn2*^fl/fl^ mice were infected with either noninvasive *E. coli* K12, or fluorescent-tagged *m*AIEC (*m*AIEC^red^) for four consecutive days or administered PBS. Subsequently, bacterial colonization in mouse tissues was quantified. mRNA and protein expression were assayed in intestinal epithelial cells (IECs) or whole tissue lysates by PCR and Western blot. Tissue cytokine expression was determined by ELISA. Intestinal barrier function was determined by *in vivo* administration of 4 kDa FITC-dextran (FD4) or 70kDa Rhodamine-B dextran (RD70) fluorescent probes. Confocal microscopy was used to determine the localization of tight-junction proteins. *Ptpn2*^∆IEC^ mice exhibited increased *m*AIEC^red^ – but not K12 – bacterial load in the distal colon compared to infected *Ptpn2*^fl/fl^ mice. The higher susceptibility to *m*AIEC^red^ infection was associated with altered levels of antimicrobial peptide (AMPs). Ileal RNA expression of the alpha-defensin AMPs, *Defa5*, and *Defa6*, as well as MMP7, was significantly lower in *Ptpn2*^∆IEC^ vs. *Ptpn2*^fl/fl^ mice, after *m*AIEC^red^ but not K12 infection. Furthermore, we observed an increased tight junction-regulated permeability determined by elevated *in vivo* FD4 but not RD70 permeability in *Ptpn2*^∆IEC^-K12 mice compared to their respective controls. This effect was further exacerbated in *Ptpn2*^∆IEC^ *m*AIEC-infected mice. Further, *Ptpn2*^∆IEC^ mice displayed lower IL-22, IL-6, IL-17A cytokine expression post *m*AIEC infection compared to *Ptpn2*^fl/fl^ controls. Recombinant IL-22 reversed the FD4 permeability defect and reduced bacterial burden in *Ptpn2*^∆IEC^ mice post *m*AIEC challenge. Our findings highlight that the intestinal epithelial PTPN2 is crucial for mucosal immunity and gut homeostasis by promoting anti-bacterial defense mechanisms involving coordinated epithelial-immune responses to restrict pathobiont colonization.

## GRAPHICAL ABSTRACT

**Figure:**
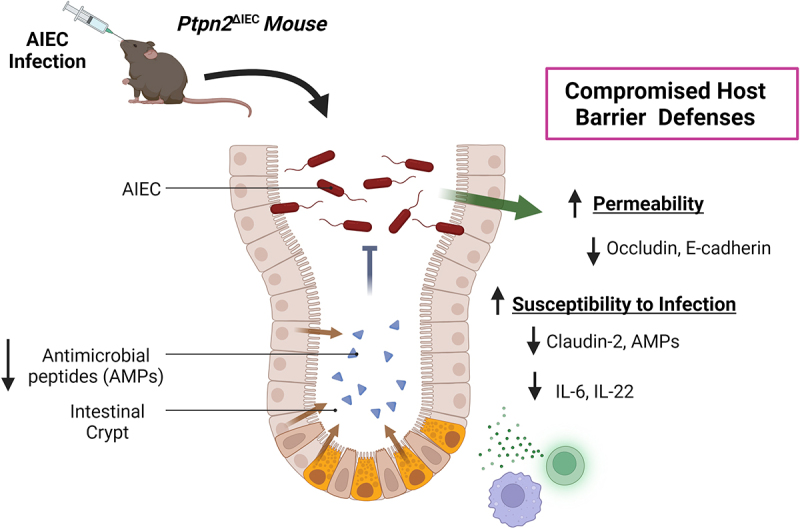

## Introduction

The intestinal epithelium has a strategic position as a physical barrier between trillions of luminal microbes and the immune cells in the underlying lamina propria while also coordinating a very delicate equilibrium to maintain mucosal homeostasis.^1–3^ Dysregulation of this physical barrier leads to very serious local and systemic consequences including gastrointestinal infections, inflammatory bowel disease (IBD), type 1 diabetes (T1D), and autoimmune arthritis.^4–6^

IBD is a chronic and multifactorial condition affecting more than 6 million people worldwide.^7,8^ IBD clinically manifests as a heterogenous disease that can broadly be classified as – Crohn’s Disease (CD) and Ulcerative colitis (UC)^9^. IBD involves a complex interplay of host genetics, gut microbiota, environmental factors, and the immune system. Genome-wide association studies (GWAS) have identified approximately 240 genes associated with IBD.^10^ One of the genes identified with disease-associated single nucleotide polymorphisms (SNP) is the protein tyrosine phosphatase non-receptor type 2 (*PTPN2*) gene that encodes the protein, T-cell protein tyrosine phosphatase (TCPTP).^10,11^ Loss of *PTPN2* function leads to aberrant activation/proliferation of T-cells and causes hyper-responsiveness of the Janus Activated Kinase (JAK)- signal transducer and activator of transcription (STAT) pathway.^12,13^ Further, significant alterations in the intestinal microbial community – dysbiosis – have been observed in IBD patients including those carrying *PTPN2* SNPs.^14,15^ One hypothesis related to the underlying pathogenesis of IBD is that genetically pre-disposed hosts have an altered intestinal environment that favors the expansion of commensal microbes with pathogenic potential, “pathobionts,” while reducing the number of protective commensal bacteria. One such pathobiont is adherent-invasive *E. coli* (AIEC), which was first isolated from the ileum of a CD patient.^16^ AIEC can adhere and attach to intestinal epithelial cells, in addition to surviving within macrophages.^17^ Previously, we have reported that whole body constitutive *Ptpn2*-KO mice exhibit a reduced abundance of *Bacteroidetes* and a greatly increased abundance of *Proteobacteria* compared to *Ptpn2* wild-type littermates. Of note, the greatest increase in abundance within the phylum *Proteobacteria* in *Ptpn2*-KO mice was a novel *Escherichia coli* species that shared significant sequence overlap with human AIEC. We have labelled this novel mouse adherent-invasive *E. coli* (*m*AIEC) strain, UCR-PP2.^18^

Intestinal epithelial cells (IECs) are physically connected to each other by tight junction (TJ), adherens junction (AJ) and desmosome protein conglomerates that maintain a tightly regulated semi-permeable barrier while supporting an intact yet flexible epithelium.^19–22^ Disruption of these proteins often leads to increased paracellular barrier permeability.^23^ The epithelium plays an essential role in the gut as it interacts with the commensal population in the luminal space and transmits specific signals that educate the underlying innate and adaptive immune cell population, thereby maintaining mucosal immune homeostasis.^24–26^ Increases in epithelial permeability or damage to the epithelial barrier can permit commensals and potentially pathogenic bacteria to interact with the immune cells in the lamina propria. Interaction with certain pathobionts can induce an unwanted inflammatory response or in more severe cases give these pathobionts access the circulation and cause sepsis. Mucosal immune cells are critical in eliminating invading pathogens as they secrete cytokines and phagocytose bacteria that are essential for pathogen-clearance^27^. Specialized IECs called Paneth cells, are also important for bacterial defenses as they secrete several antimicrobial peptides that kill bacteria or inhibit microbial growth.^28,29^ Therefore, the intestinal barrier is essential for mucosal homeostasis in part by restricting the increase in intestinal permeability that is an early and critical event in the development of several inflammatory conditions including IBD.^30,31^ Previously, our lab has demonstrated that *Ptpn2*-KO mice display increased intestinal permeability and reduced antimicrobial peptide production.^32,33^ These results point toward epithelial *Ptpn2* as an important contributor to maintenance of the intestinal barrier and antimicrobial defenses.

However, there remains a major gap in our understanding of how host genetics alters the gut microbial landscape and how opportunistic pathobionts subvert the host defense machinery and manifest complex conditions in a disease susceptible host. Thus, the main aim of this study was to determine the role of *Ptpn2* in the intestinal epithelium in mediating microbiome-immune cell crosstalk to prevent *m*AIEC colonization and preserve gut homeostasis.

## Methods

### Animal procedures

#### Ethical statement on mouse studies

All animal care and procedures were performed in accordance with institutional guidelines and approved by the University of California, Riverside Institutional Animal Care and Use Committee under Protocol #A2022001B.

### Housing and husbandry of experimental animals

Tamoxifen-inducible IEC-specific *Ptpn2* knockout (*Ptpn2* ^∆IEC^) was generated as previously described.^33^ We also confirmed IEC-specific knockout in *Ptpn2* ^∆IEC^ mice via immunostaining and western blotting.^33^ The mice were housed in a temperature-controlled room with a 12/12 hour light and dark cycle under specific-pathogen-free conditions at the University of California, Riverside (UCR). The mice had access to normal chow and water *ad libitum*. Five to six-week-old male and female mice were injected with tamoxifen (Sigma-Aldrich, Saint Louis, MO) via intraperitoneal injections at 1 mg/mL in 100 μl of corn oil for 5 consecutive days. Twenty-eight-days after the final injection, mice were randomly allocated to be oral gavaged with 100 μl of either PBS, *E. coli* K12 or *m*AIEC^red^ (*m*AIEC strain-UCR PP2) at 10^9^ cfu/mL for 4 consecutive days. Mice were injected intraperitoneally with recombinant IL-22 (Peprotech, Thermo Fisher Scientific, NJ) at 20 ug/mL per mice every 48 h. Bacterial infections were performed as described above. Euthanasia was performed via isoflurane at the end of the experimental study.

### Bacterial infection and immunofluorescence studies

Bacteria from stocks frozen at −80°C in 1:1 vol/vol glycerol: LB were cultured overnight in Luria – Bertani (LB) broth supplemented with chloramphenicol at 37°C, 250–300 rpm and regrown the next day in fresh LB-chloramphenicol to exponential phase growth. Culture was pelleted, washed with phosphate buffered saline (PBS) and resuspended in PBS. The bacteria used were *m*AIEC^red^, and K12 (a noninvasive *E. coli*, ATCC 25404).

### Tissue RNA isolation and quantitative PCR

Total RNA was extracted from intestinal segments of mice using RNeasy Mini kit (Qiagen, Hilden, Germany). RNA purity and concentration were assessed by absorbance at 260 and 280 nm. One microgram of total RNA was transcribed into cDNA using qScript cDNA SuperMix (Quanta Biosciences, Beverly, MA). Two microliters of 5×-diluted cDNA were amplified using gene-specific primers (Supplementary Table S1) and GoTaq Green, 2× mix. Gene-specific primers were used with the following conditions: initial denaturation 95°C for 5 min, followed by 30 cycles 95°C for 30 s (denaturation), 55°C for 30 s (annealing), and 72°C for 30 s (extension). The final extension was 72°C for 5 min. Mouse GAPDH was used as an endogenous control.

### In-vivo barrier permeability

Mice were gavaged with 80 mg/mL of fluorescein isothiocyanate (FITC)-dextran (4 kDa) (Sigma-Aldrich) and 20 mg/mL of rhodamine B-dextran (70 kDa) (Sigma-Alrich). After 5 h, blood was collected by retro-orbital bleed into serum collection tubes. Blood was centrifuged at 4°C, 1500 *g*, for 15 min, and serum was analyzed for FITC-dextran and rhodamine B-dextran concentrations. Fluorescence of FITC and rhodamine in samples was determined by loading serum into a black plate and measuring excitation wavelengths of 495 nm and 555 nm, and emission wavelengths of 525 nm and 585 nm, respectively, using a SpectraMax iD3 plate reader (Molecular Devices, San Jose, CA), aligned with established protocols.^33,34^ Standard curves for calculating fluorophore concentration in the samples were obtained by diluting the fluorophore stock in sterile MilliQ.

### Isolation of IECs and western blot analysis

Intestinal tissues from the ileum or proximal colon were everted and incubated in Cell Recovery Solution (Corning, New York) on ice for 2 h, then vigorously shaken by hand to release IECs. IECs were washed twice with ice-cold PBS, then lysed with radioimmunoprecipitation assay (RIPA) buffer (50 mM Tris-HCl pH 7.4, 150 mM NaCl, 1% NP-40, 0.5% sodium deoxycholate, and 0.1% SDS) supplemented with 1× protease inhibitor (Roche, Basel, Switzerland), 2 mM sodium fluoride, 1 mM PMSF, and phosphatase inhibitors for at least 10 min on ice. Cells were homogenized on ice using the Q125 Sonicator (Newtown, CT) lysates centrifuged at 16,200*g* at 4°C for 10 min, and supernatants collected into new microcentrifuge tubes. Protein concentration was determined using the Pierce BCA Protein Assay Kit (Thermo Fisher Scientific, Waltham, MA). Loading samples were prepared by mixing the same amount of total protein from each sample, with Laemmli loading buffer (60 mM Tris-HCl pH 6.8, 2% SDS, 5% β-mercaptoethanol, and 10% glycerol), then boiling the samples at 95°C for 10 min. Twenty µg of protein was loaded on polyacrylamide gels, and after separation by gel electrophoresis, transferred onto polyvinylidene difluoride membranes. Nonspecific epitopes were blocked with 5% milk in Tris-buffered saline with 0.1% Tween-20 added for 1 h at room temperature. Membranes were incubated overnight with primary antibody at 4°C, washed (×3) with Tris-buffered saline with 0.1% Tween-20, and incubated with horseradish-peroxidase – conjugated secondary antibody against the primary antibody species (Suplementary Table S2) for 1 h at room temperature. Immunoreactive proteins were detected with x-ray films (Labscientific, Inc, Highlands, NJ) using the SuperSignal West Pico PLUS chemiluminescence detection kit (Thermo Fisher Scientific, Waltham, MA).

### Enzyme-linked immunosorbent assay (ELISA)

Colonic whole tissue was excised to perform ELISAs. IL-22 and IL-6 DuoSet enzyme-linked immunosorbent assays were obtained from R&D Systems (Minneapolis, MN) and performed according to the manufacturer’s guidelines.

### Immunofluorescence

Mouse intestinal segments were embedded in OCT and frozen sections cut into 5-μm-thick sections. Slides were brought to room temperature, rinsed twice with PBST (PBS +0.1% Tween20), and fixed in methanol (10 min at −20°C). Slides were then incubated in blocking buffer (PBS +2% donkey serum, 1% BSA, 1% Triton X, 0.05% Tween-20) for 1 hour before overnight incubation with F’ab donkey-anti-mouse antibody (Jackson ImmunoSearch Inc., Jackson, PA). The slides were rinsed with PBST, incubated with primary antibody (diluted in PBS + 5% normal donkey serum) for 30 min at room temperature, rinsed again with PBS before incubation with a biotinylated secondary anti-mouse antibody (Jackson ImmunoSearch Inc.) for 20 min, followed by an additional wash in PBST and subsequent incubation with Alexa Fluor 488-steptavidin, Alexa Fluor 647 secondary antibody (Jackson ImmunoSearch) for 30 min, and finally mounted with ProlongGold Antifade Reagent with DAPI (Thermo Fisher Scientific). Images were acquired with an inverted Zeiss 880 Airy-scan confocal microscope.

### Statistical analyses

Critical significance was set at α = 0.05. The data were analyzed for parametric distribution by Shapiro–Wilk test. Data are expressed as mean ± SD for *n* number of biological replicates. Between-group inferences were made by using 1-way or 2-way analysis of variance (ANOVA) followed by Tukey’s post-hoc test. All statistical analyses were performed in GraphPad Prism version 10 (GraphPad Inc., La Jolla, CA).

## Results

### Loss of intestinal epithelial PTPN2 promotes mAIEC colonization

**Figure 1.**
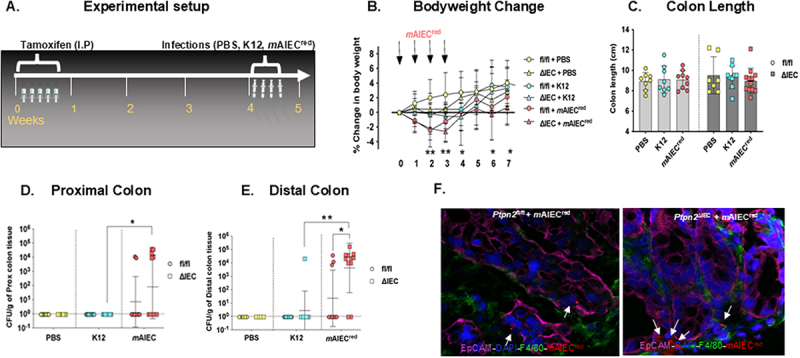
Loss of intestinal epithelial *Ptpn2* promotes *m*AIEC colonization.

Previous studies from our lab showed that constitutive whole-body *Ptpn2*-deficient mice exhibit expansion of a novel *m*AIEC and display reduced expression of antimicrobial peptides.^18,32^ To determine the specific role of epithelial *Ptpn2* in limiting *m*AIEC colonization, we used a tamoxifen-inducible *Ptpn2* Villin-cre transgenic mouse model, which either carried the epithelial floxed *Ptpn2* gene (*Ptpn2*^fl/fl^) but was Cre-negative, or the Cre-positive specific deletion of the epithelial *Ptpn2* gene (*Ptpn2*^∆IEC^). The IEC-specific deletion of PTPN2 has been previously confirmed in our model via immunohistochemistry and western blotting.^32^ The mice were treated with PBS; a nonpathogenic, noninvasive *E. coli*, K12; or mCherry fluorescent-tagged *m*AIEC^red^ (Figure 1(A)). We used *E. coli* K12 as a bacterial control to distinguish specific host responses to bacterial colonization between a noninvasive *E. coli* and the invasive AIEC strain of bacteria. We validated *m*AIEC^red^ adherence and invasion to intestinal epithelial cells (Supplementary 1(A,B,D)) and its colonization persistence in mice (Supplementary Figure S1(C)). *m*AIEC^red^ infected mice displayed a mild decrease in body weight in both *Ptpn2* ^∆IEC^ and *Ptpn2* ^fl/fl^ mice (Figure 1(B)), while no changes in colon length were observed (Figure 1(C)). We also observed a trend toward higher colonization of *m*AIEC^red^ in the proximal colon (Figure 1(D)). Interestingly, we observed, *Ptpn2* ^∆IEC^ had higher *m*AIEC^red^ burden in the distal colon tissue compared to *Ptpn2* ^fl/fl^ mice (Figure 1(E)). Expectedly, *m*AIEC^red^ displayed higher proximal colon colonization in *Ptpn2* ^∆IEC^ mice compared to its K12 infected counterparts (Figure 1(D,E)). Furthermore, we saw no difference in bacterial load of *E. coli* or *m*AIEC in the luminal contents between the two genotypes (Supplementary Figure S2(A-D)) suggesting that while the luminal bacterial *m*AIEC load remained similar between the genotypes, *Ptpn2*^∆IEC^ mice were more susceptible to *m*AIEC colonization of IECs. The increased colonization of *Ptpn2*^∆IEC^ was visually confirmed by immunofluorescence staining. Higher bacterial burden was associated with epithelial cells stained with epithelial cellular adhesion molecules (EpCam) as compared to the macrophages stained with ADGRE1 (F4/80) (Figure 1(F)). Taken together, these data demonstrate that IEC-specific loss of *Ptpn2* increases host susceptibility to *m*AIEC infection.

*Ptpn2*^fl/fl^ or *Ptpn2*^∆IEC^ were given (A) tamoxifen at 1 mg/mL for 5 consecutive days. After a period of 28 days, the mice were given PBS, *E. coli* K12 or *m*AIEC^red^ (10^9^ bacteria per mouse in 100 ul of PBS) from day 0-3 (*n* = 9–12). The mice were euthanized at day 7. (B) *m*AIEC^red^ infected groups display mild loss of body weight (*P* = 0.006). (C) Colon length was measured. Bacterial colonies were enumerated from (D) proximal colon whole tissues (*Ptpn2*^∆IEC^ - *m*AIEC^red^ mice vs. *Ptpn2*^∆IEC^ – K12 mice) (*P* = 0.0194) and (E) distal colon (*Ptpn2*^∆IEC^ - *m*AIEC^red^ mice vs. *Ptpn2*^fl/fl^ – *m*AIEC) (*p = 0.002)*. (F) Immunofluorescence imaging of *Ptpn2*^fl/fl^ or *Ptpn2*^∆IEC^ proximal colon section infected with *m*AIEC^red^. Epithelial cells were marked in EpCam (magenta), macrophages were marked by F4/80 (green) and *m*AIEC^red^ in red. Results are a combination of three independent experiments. The normal distribution is assessed by Shapiro−Wilk test. Groups were compared with two-way AN OVA with Tukey multiple comparison test. *P < 0.05, **p < 0.01.

### Epithelial PTPN2 restricts bacterial induced intestinal barrier permeability

**Figure 2.**
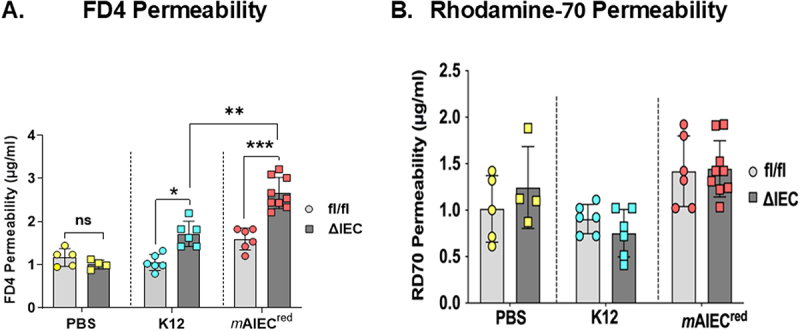
*Ptpn2* ^_^ deficient epithelial cells display increased intestinal barrier permeability.

Previous studies in our lab have shown that *m*AIEC infection exacerbated permeability increases in wildtype C57Bl/6 mice co-challenged with dextran sodium sulfate (DSS) to induce colitis post infection.^18^ Therefore, we wanted to determine if AIEC challenged *Ptpn2*^∆IEC^ can exert an effect in intestinal barrier permeability. We assessed *in vivo* FD4 and RD70 permeability after a bacterial challenge. Consistent with our previous data, no significant differences in FD4 permeability were observed between *Ptpn2*^fl/fl^ and *Ptpn2*^∆IEC^ groups that were treated with PBS (Figure 2(A)). *E. coli* K12 increased FD4 permeability in *Ptpn2*^∆IEC^ mice compared to their floxed controls, while *m*AIEC^red^ caused an even greater increase in FD4 permeability in *Ptpn2*^∆IEC^ versus *Ptpn2*^fl/fl^ mice (Figure 2(A)) with a 175 ± 48% (“delta”) increase (mean ± SD) compared to K12 which caused a 77 ± 33% increase in permeability relative to PBS treated *Ptpn2*^∆IEC^ mice (*p* = 0.0087). Moreover, no significant difference was observed in RD70 permeability between *Ptpn2*^fl/fl^ and *Ptpn2*^∆IEC^ mice, or between treatments, suggesting that the epithelial lining was not functionally damaged (Figure 2(B)). Overall, these data demonstrate that the loss of epithelial PTPN2 increases FD4 permeability after bacterial infection, increasing the tight junction-regulated paracellular barrier permeability but not nonspecific permeability arising from damage to the epithelium.

Intestinal barrier permeability was measured by (A) FITC -Dextran Permeability (*Ptpn2*^∆IEC^ -K12 vs *Ptpn2*^fl/fl^ -K12 controls (*P* = 0.0025) and *Ptpn2*^∆IEC^ - K12 vs *Ptpn2*^∆IEC^ - *m*AIEC (*P* = 0.0067)) and (B) rhodamine permeability. Results are a combination of two independent experiments. Normal distribution was assessed by Shapiro–Wilk test. Groups were compared with two-way ANOVA and Tukey multiple comparison tests. *P < 0.05, **P < 0.01, P***0.001.

### AIEC induces greater disruption of barrier-forming proteins in mice lacking PTPN2 in intestinal epithelial cells

**Figure 3.**
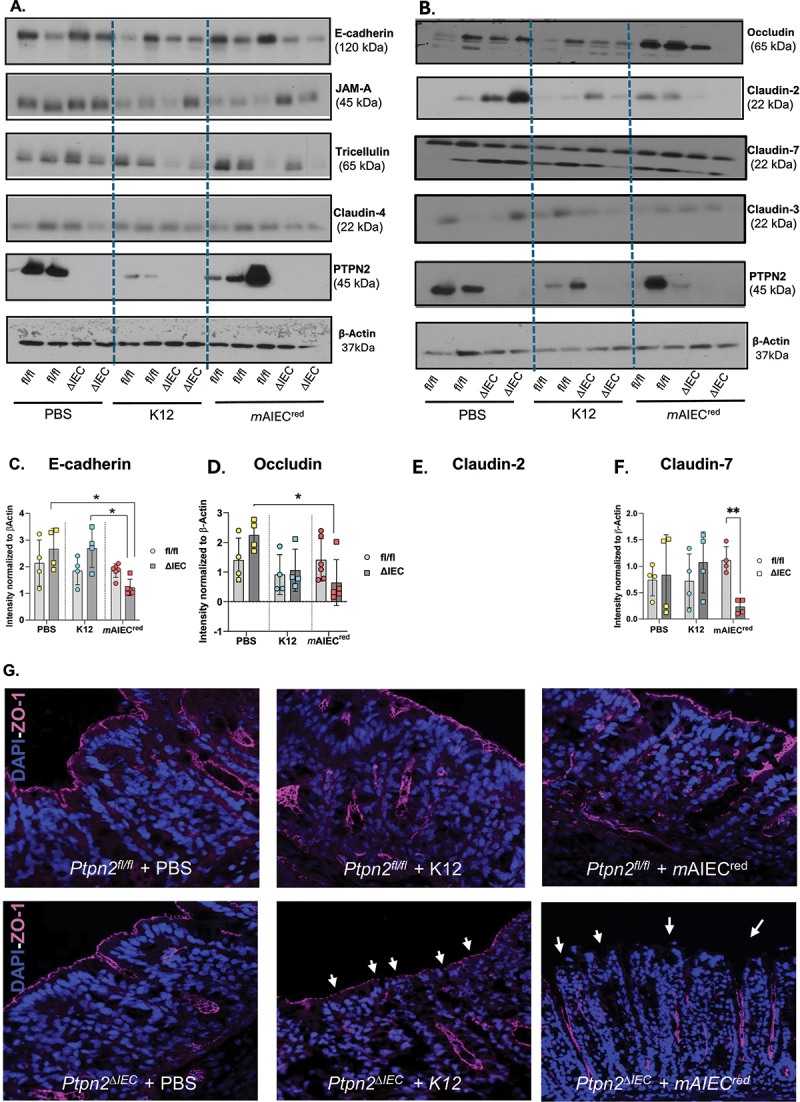
*Ptpn2* ^_^ deficient epithelial cells display alterations in barrier-forming proteins post-bacterial infection.

Next, we investigated if AIEC-induced changes in intestinal permeability in *Ptpn2*^∆IEC^ mice were associated with alterations in barrier-forming proteins. In line with the FD4 results, proximal colon IECs isolated from *Ptpn2*^∆IEC^ mice infected with *m*AIEC^red^ had decreased E-cadherin and occludin protein levels compared to their PBS-treated *Ptpn2*^∆IEC^ counterparts, while *m*AIEC^red^ challenge also significantly reduced E-cadherin expression compared to K12 challenge in *Ptpn2*^∆IEC^ mice (Figure 3(A-D)). However, no change was observed in E-cadherin or occludin expression between floxed vs. *Ptpn2*^∆IEC^ mice challenged with *m*AIEC^red^ even though these conditions did exhibit a significant difference in FD4 permeability (*c.f*. Figure 2(A)). Other barrier-forming proteins like JAM-A and tricellulin remain unchanged between genotypes or treatment groups (Figure 3(A)). Further, we determined the levels of the claudin family of proteins which are essential for effective barrier function. Claudin-2 is a cation pore-forming member of the claudin family of transmembrane proteins that is frequently elevated during inflammation, including in IBD, while during bacterial infection its expression is increased as part of a host-protective mechanism.^35,36^ Consistent with our previous studies, claudin-2 levels were significantly elevated in *Ptpn2*^∆IEC^ mice compared to the *Ptpn2*^fl/fl^- PBS treated littermates, while a similar, albeit not statistically significant, effect was observed in the K12 infected *Ptpn2*^∆IEC^ mice (Figure 3(B,E)). Strikingly, the increase in claudin-2 levels in *Ptpn2*^∆IEC^ was reversed after *m*AIEC infection, with these mice displaying a significant reduction in claudin-2. This suggests that *m*AIEC can suppress expression of critical host-protective mechanisms, like claudin-2, even in conditions where claudin-2 is overexpressed (Figure 3(B,E)). Protein levels of the barrier-enhancing protein, claudin-7, were also reduced in *Ptpn2*^∆IEC^ – *m*AIEC group compared to their respective controls (Figure 3(B,F)). The claudin-3 and 4 protein levels remained unchanged by genotype or treatment (Figure 3(A,B)). Localization of the tight junction regulatory protein, ZO-1, remained intact in all the *Ptpn2*^fl/fl^ groups; however, small gaps were seen in *Ptpn2*^∆IEC^ – K12 group (Figure 3(G)). Furthermore, ZO-1 localization was completely ablated in the *Ptpn2*^∆IEC^ – *m*AIEC group compared to controls (Figure 3(G)).

(A), (B). Representative western blot images for E-cadherin, occludin, JAMA, tricellulin, claudin-2,3,4 and 7. Densitometry analysis of (C) E-cadherin (*Ptpn2*^∆IEC^ - *m*AIEC^red^ vs. *Ptpn2*^∆IEC^- PBS (*P* = 0.0124) and *Ptpn2*^∆IEC^- K12 (*P* = 0.0142). (D) Occludin (*Ptpn2*^∆IEC^ - *m*AIEC^red^ vs. *Ptpn2*^∆IEC^- PBS controls (*P* = 0.0042)). (E) Claudin-2 *(Ptpn2*^∆IEC^- *m*AIEC^red^ mice vs. *Ptpn2*^fl/fl^ -*m*AIEC^red^ controls (*P* = 0.004)) (F) Claudin-7 (*Ptpn2*^∆IEC^ - *m*AIEC^red^ vs. *Ptpn2*^fl/fl^- *m*AIEC^red^ (*P* = 0.0009)). Immunofluorescence staining of proximal colon whole tissue. (G) Immunofluorescence staining of ZO-1 (magenta) in *Ptpn2*^fl’fl^ and *Ptpn2*^∆IEC^ proximal colon tissues. Gaps are indicated by arrows. Normal distribution was assessed by Shapiro–Wilk test. Groups were compared with two-way ANOVA with Tukey multiple comparison tests. *P < 0.05, **P < 0.01, P***0.001.

### PTPN2 deficiency results in reduced antimicrobial peptide production in response to mAIEC infection

**Figure 4.**
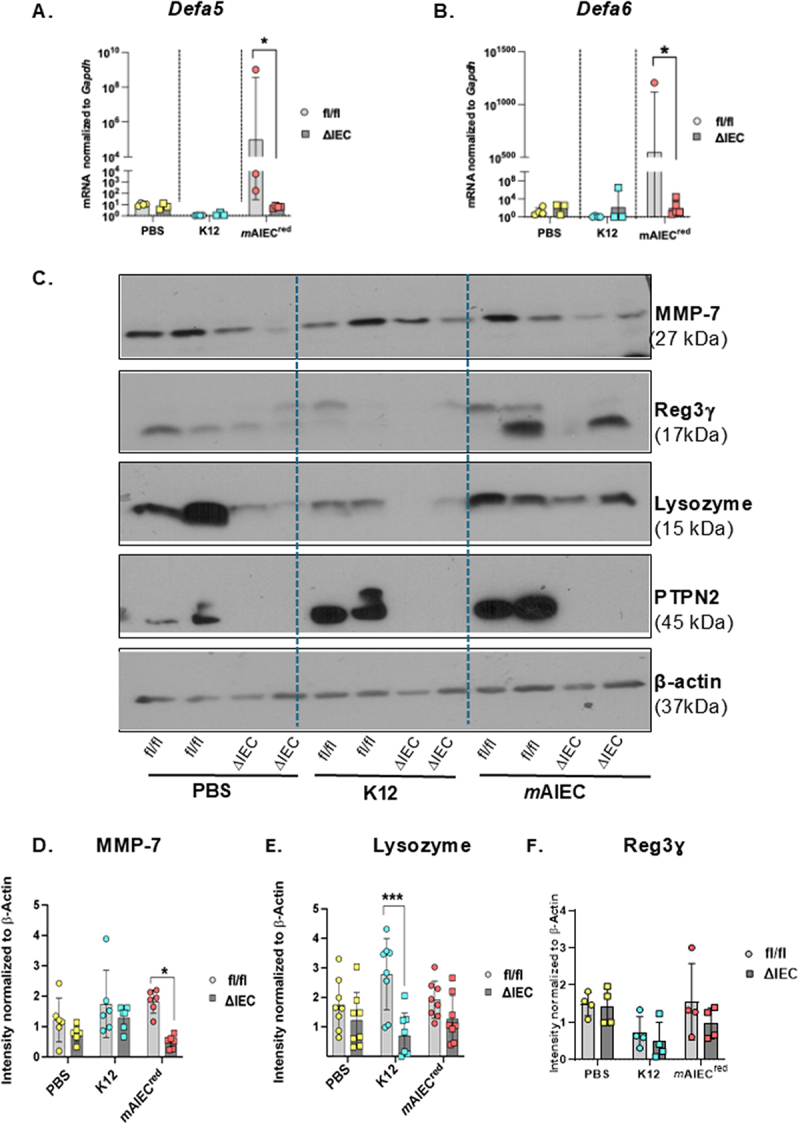
*Ptpn2* deficiency results in reduced AMP production in response to mAIEC infection.

Given that *Ptpn2*^∆IEC^ mice exhibited higher *m*AIEC^red^ bacterial load in distal colon tissue, and our previous observations that whole-body constitutive *Ptpn2*-KO mice have reduced antimicrobial peptide (AMP) production and numbers of Paneth cells, we next investigated if the increased susceptibility of *Ptpn2*^∆IEC^ mice to *m*AIEC^red^ infection involved altered host antimicrobial peptide defenses.^32^ Ileal IEC-mRNA expression of the ***α***-defensins (*Defa5* and *Defa6*), was significantly lower in *Ptpn2*^∆IEC^ vs. *Ptpn2*^fl/fl^ mice, after *m*AIEC^red^ but not K12 infection (Figure 4(A,B)). Furthermore, we found that the ileal IEC protein levels of the AMP, lysozyme, were significantly lower in K12 infected *Ptpn2*^∆IEC^ compared to its respective control littermates (Figure 4(C,E)). However, the same effect was not seen in the PBS or *m*AIEC infected groups (Figure 4(A,E)). No difference was observed in levels of regenerating islet-derived 3 gamma (Reg3ɣ), between genotypes or treatments (Figure 4(C,F)). In addition, the expression of the protease matrix metalloproteinase-7 (MMP-7) – which is responsible for the proteolytic cleavage and activation of α-defensins – was significantly decreased in the ileum of *Ptpn2*^∆IEC^ mice post *m*AIEC^red^ infection compared to *Ptpn2*^fl/fl^ littermates (Figure 4(C,D)). Together, these data demonstrate that intestinal epithelial PTPN2 is critical for AMP-mediated defenses in response to *m*AIEC infection.

Antimicrobial peptide expression was measured from ileum whole tissues. (A) *Defa5* (*Ptpn2*^∆IEC^ - *m*AIEC^red^ vs. *Ptpn2*^fl/fl^ - *m*AIEC^red^ (*P* = 0.0008)) (B) *Defa6 (Ptpn2*^∆IEC^ - *m*AIEC^red^ vs. *Ptpn2*^fl/fl^ - *m*AIEC^red^ (*P* = 0.00168)). (C) Representative western blot images for MMP-7, Reg3ɣ and Lysozyme from ileum IECs. Densitometry analysis of (D)MMP-7 (*Ptpn2*^∆IEC^ - *m*AIEC^red^ vs. *Ptpn2*^fl/fl^ - *m*AIEC^red^ mice (*P* = 0.042). (E) Lysozyme (F) Reg3ɣ. Normal distribution was assessed by Shapiro−Wilk test. Groups were compared with 2-way ANOVA with Tukey multiple comparison tests. *P < 0.05, **P < 0.01, P***0.001.

### Intestinal epithelial PTPN2 deficiency caused reduced cytokine expression in response to mAIEC infection

**Figure 5.**
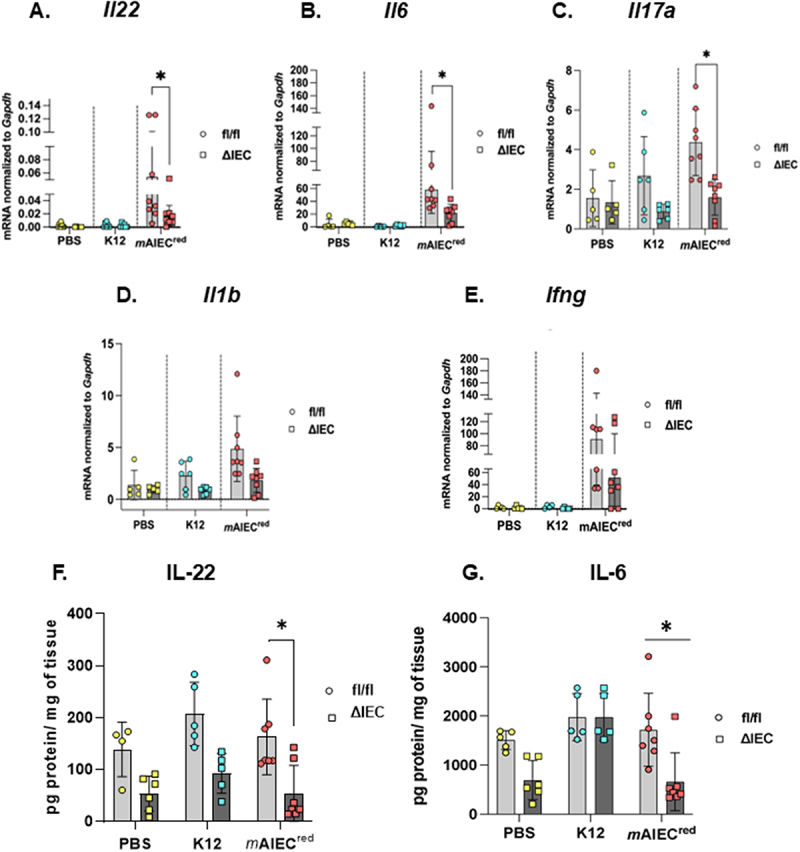
*Ptpn2* deficiency results in reduced cytokine expression in response to mAIEC infection.

Next, we determined the expression profiles of mucosal cytokines involved in promoting Paneth cell function and clearance of bacteria and bacterial products. Typically, T-helper cells 1 (Th1) mount a cytokine reaction during bacterial invasion which is more inflammatory in nature, whereas the T-helper cells 2 (Th2) mount a response against parasitic infestations. Additionally, mucosal cytokines are important in intestinal barrier maintenance. We observed mRNA expression of *Il22*, *Il6*, and *Il17* was significantly reduced in the proximal colon of *Ptpn2*^∆IEC^ - *m*AIEC^red^ mice compared to their control littermates (Figure 5(A-C)). Interferon-gamma (*Ifng*) and *1l1b* levels remained similar between genotypes and treatments (Figure 5(D,E)). As a known repressor of *Il22* transcription, we also probed for p-STAT1 in our model. We noticed that p-STAT1 levels are elevated in both naïve and infected *Ptpn2*^∆IEC^ mice (Supplementary Figure S3(D)). Further, western blotting on proximal colonic whole tissues identified reduced expression of the general immune cell marker, cluster of differentiation-45 (CD45), and the T-cell marker, CD3 in *Ptpn2*^∆IEC^ – *m*AIEC mice in comparison with their respective controls (Supplementary Figure S3(A-C)). Further, no changes were observed in expression of Type 2 cytokines *Il4*, *Il13* or the Th-2 cell marker, GATA-binding protein 3 marker (*Gata3*) (Supplementary Figure S4(A-C)). Next, we validated the mRNA data by confirming that in colonic whole tissues, PBS treated *Ptpn2*^∆IEC^ mice had reduced IL-22 and IL-6 protein in comparison to its *Ptpn2*^fl/fl^ control, as determined by ELISA (Figure 5(F,G)). These data indicate that protective barrier and AMP-promoting cytokine responses are impaired in *Ptpn2*^∆IEC^ mice.

Cytokine expression profile was measured from proximal colon whole tissue sample. (A) *Il22* (*P* = 0.0234) (B) *Il6* (*P* = 0.0208) (C) *Il17a* (*P* = 0.00216) (D)*Ifng* and (E) *Il1b*. ELISA was performed from whole-tissue distal colon samples. Protein levels of (F) IL-22 (*Ptpn2*^∆IEC^ - *m*AIEC^red^ vs. *Ptpn2*^fl/fl^ - *m*AIEC^red^ (*P* = 0.0012) and (G) IL-6 (*P* = 0.005) was measured. Outliers were analyzed by Grubb’s test. Normal distribution was assessed by Shapiro−Wilk test. Groups were compared with two-way AN OVA with Tukey multiple comparison tests. *P < 0.05, **P < 0.01, P***0.001.

### Recombinant IL-22 reduces mAIEC bacterial load and ameliorates the FD4 permeability defect in Ptpn2^∆IEC^ mice in response to mAIEC infection

**Figure 6.**
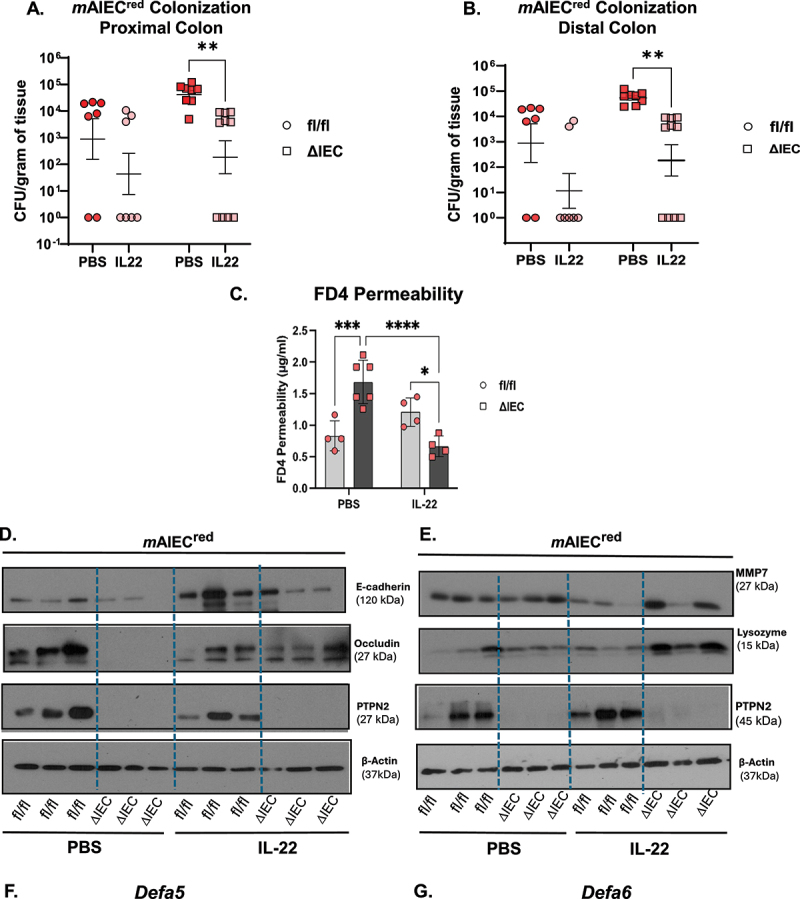
Recombinant IL-22 reduces mAIEC bacterial load and ameliorates FD4 permeability defect in *Ptpn2* deficient mice in response to mAIEC infection.

Given that IL-22 plays a critical role in the production of AMPs and is also essential for the integrity of the mucosal barrier, we next examined if reconstitution of IL-22 in *Ptpn2*^∆IEC^ mice augmented host defenses to limit *m*AIEC^red^ colonization and restore epithelial barrier function. Vehicle-treated *Ptpn2*^∆IEC^ mice (+ *m*AIEC) displayed higher *m*AIEC CFU burden in the proximal and the distal colon compared to *Ptpn2*^fl/fl^ mice treated with vehicle alone, whereas IL-22 administration in both *Ptpn2*^fl/fl^ and *Ptpn2*^∆IEC^ mice significantly reduced the *m*AIEC^red^ CFU burden (Figure 6(A,B)). Next, we determined differences in FD4 permeability between vehicle and recombinant IL-22 treated *Ptpn2*^fl/fl^ and *Ptpn2*^∆IEC^ mice post *m*AIEC infection. We also observed that FD4 permeability in IL-22 administered *Ptpn2*^∆IEC^-*m*AIEC^red^ mice was significantly reduced compared to the vehicle-treated controls (Figure 6(C)). Interestingly, the same effect was not observed between the vehicle or recombinant IL-22 treated *Ptpn2*^fl/fl^-*m*AIEC group (Figure 6(C)). Next, we examined the levels of tight junction proteins that were previously diminished post *m*AIEC^red^ challenge. We observed that protein levels of both occludin and E-cadherin were restored after IL-22 administration in *Ptpn2*^∆IEC^-*m*AIEC^red^ group (Figure 6(D)). Further, ileal IECs displayed elevated levels of MMP7, and lysozyme in *Ptpn2*^∆IEC^-*m*AIEC^red^ group after administration of IL-22 (Figure 6(E)). Additionally, antimicrobial molecules *Defa5* and *Defa6* were also significantly higher in the *Ptpn2*^∆IEC^-*m*AIEC^red^ group reconstituted with IL-22 in comparison with the PBS controls (Figure 6(F,G)). These data indicate that increased colonization and the subsequent barrier defect post *m*AIEC^red^ infection in *Ptpn2*^∆IEC^ mice were reversed by IL-22 supplementation.

Recombinant IL-22 or PBS was administered in *Ptpn2*^fl/fl^ or *Ptpn2*^∆IEC^ mice prior to *m*AIEC^red^ infection and subsequently injected every 48 h till the mice were sacrificed. *m*AIEC^red^ tissue burden was enumerated from (A) Proximal colon (*p=*0.0092) and (B) Distal colon (*p=*0.0096). (C) FD4 permeability was measured in PBS and IL-22 treated mice post-*m*AIEC infection (*p=*0.0002). Representative Western blot images of (D) E-cadherin and occludin levels in IL-22 and PBS administered *Ptpn2*^fl/fl^ or *Ptpn2*^∆IEC^ Respresentative Western blot images of (E) MMP7 and lysozyme levels in ileal IECs of *Ptpn2*^∆IEC^ and *Ptpn2*^fl/fl^ – *m*AIEC after reconstitution of IL-22. Transcript levels of (F) *Defa5* and (G) *Defa6* were measured fin ileal IECs (*p=*0.0026; *p=*0.00134). Results are a combination of two independent experiments. Normal distribution was assessed by Shapiro–Wilk test. Groups were compared with two-way AN OVA with Tukey multiple comparison tests. *P < 0.05, **P < 0.01, P***0.001.

## Discussion

PTPN2 modulates intestinal microbial composition, antimicrobial peptide levels, and mucosal barrier permeability.^18,32,33^ We have previously demonstrated that constitutive whole-body loss of *Ptpn2* results in expansion of AIEC and reduction in Firmicutes such as segmented filamentous bacteria (SFB).^18^ Furthermore, we have demonstrated that loss of *Ptpn2* is critical for regulation of intestinal permeability through epithelial-macrophage crosstalk and preservation of barrier-forming proteins.^37^ In this study, we demonstrate for the first time that intestinal epithelial PTPN2 is critical for immunity against *m*AIEC infection by promoting antimicrobial defense molecules, maintaining barrier integrity, and coordinating protective immune cell-mediated cytokine responses, highlighting the central role of PTPN2 in microbial-epithelial-immune homeostasis.

We confirmed that our fluorescent AIEC (*m*AIEC^red^) has similar properties of adherence, invasion, and survival in macrophages compared to its human clinically relevant counterpart.^38^ Further, *m*AIEC^red^ can localize in IECs *in vivo* which validates its identity as an invasive bacterium. While the first AIEC was discovered in ileal tissues of a Crohn’s disease patient, several other AIEC strains have been discovered from other intestinal regions.^16,39–41^ *m*AIEC is present in low levels in wild-type mice but displayed much higher abundance in constitutive *Ptpn2*-KO mice in both the small and large intestine.^18^ Of note, in this current study *m*AIEC^red^ preferentially colonized the distal colon versus more proximal regions of the intestine. While the reasoning for this has not yet been determined, it may reflect the impact of specific bacterial niches present in different regions of the intestine, with *m*AIEC achieving the greatest colonization efficiency in the distal colon.^18^

Paneth cells are specialized secretory cells, producing several AMPs that prevent bacterial colonization and growth. Paneth cells are localized in the ileum. However, Paneth cell-derived ***α***-defensins is abundantly found in the colonic lumen and display identical bactericidal activity as the α-defensins of the ileum. In the clinic, CD patients present with Paneth cell abnormalities with reduced expression of α- defensins.^42^ In this study, we observed that *Ptpn2*^∆IEC^ mice display reduced expression of the α-defensins, *Defa5* and *Defa6*, in response to *m*AIEC. Furthermore, these mice also display loss of MMP-7, a protease that is important for activation of the defensin family of proteins, and thus also serves as a Paneth cell marker.^43^ Various studies have indicated that another class of defensins namely, the β-defensins – secreted by enterocytes in both the large and the small intestines constitutively and in response to infection – play a critical role in the colon. Interestingly, we did not find differences in β-defensins transcript levels between genotype or infection groups. Furthermore, our group has previously shown that naïve *Ptpn2*^∆IEC^ mice have lower lysozyme production compared to *Ptpn2*^fl/fl^ mice. Therefore, the data presented in this manuscript, coupled with our previously published findings, strongly suggest that *Ptpn2* has a central role in Paneth cell secretion of AMPs and limiting pathobiont colonization.

It is also important to note that Paneth cell AMPs exert important effects not just in the ileum, but their reduced expression can also exert effects more distally and disruption of ileal Paneth cells can provoke dysbiosis in the colon.^44^ This may contribute to the region-specific variations in bacterial niches along the lower intestine and the colonization preference of *m*AIEC in the distal colon.

Enhanced intestinal barrier permeability is a risk factor for onset of IBD.^45,46^ Newer evidence has demonstrated that healthy first-degree relatives of patients with CD have significantly higher risk of developing the disease if they are presented with increased intestinal permeability, suggesting that barrier loss is an early and crucial event in disease pathogenesis.^30,31^ Since the intestinal epithelium is semi-permeable, it maintains flux across the epithelial barrier through tight junction-dependent paracellular “pore” and “leak” pathways. The “unrestricted pathway” is generated by epithelial damage due to apoptosis or cell shedding. While the unrestricted pathway – assessed by RD70 – remained unchanged in our study regardless of PTPN2 genotypes or following bacterial challenge, we did observe increased paracellular flux via the leak pathway, as assessed by *in vivo* FD4 challenge. There was no observable difference in *in vivo* FD4 flux between the genotypes in the PBS condition. This was, consistent with our previous reports, although we did find region-specific increases in FD4 permeability in *ex vivo* using chamber studies indicating a mild underlying paracellular flux in *Ptpn2*^∆IEC^ mice that was exacerbated by the cytokine challenge.^33^ In our current study, we did make the intriguing finding that FD4 permeability was higher in the *Ptpn2*^∆IEC^ mice infected with either K12 or *m*AIEC compared to infected *Ptpn2*^fl/fl^ mice. This indicates that loss of PTPN2 in intestinal epithelium changes the dynamics of epithelial interactions with commensal bacteria and appears to confer increased pathobiont potential on both invasive and noninvasive commensals. This is significant as it indicates that in the background of a host genetic defect, “benign” bacteria can provoke defects in intestinal barrier function.

Functional changes in permeability following AIEC infection aligned with altered expression and localization of apical tight junction proteins. We observed that *m*AIEC infection of *Ptpn2*^∆IEC^ mice decreased levels of the transmembrane tight junction protein, occludin, and the adherens junction protein, E-cadherin compared to *Ptpn2*^∆IEC^ + PBS controls, however the effect of *m*AIEC was not significantly different between mouse genotypes. ZO-1 membrane localization was ablated in the *Ptpn2*^∆IEC^- *m*AIEC group compared to *Ptpn2*^fl/fl^ - *m*AIEC mice, while *Ptpn2*^∆IEC^ – K12 mice displayed gaps in ZO-1 localization. It is therefore possible that the differences in FD4 permeability in the *Ptpn2*^∆IEC^ - *m*AIEC and *Ptpn2*^fl/fl^ - *m*AIEC mice may be driven by reduced ZO-1 apical membrane localization. Of note, the collective changes in junctional protein expression or localization are consistent with the corresponding higher FD4 permeability observed in *Ptpn2*^∆IEC^ mice. Furthermore, the pore-forming, tight junction protein, claudin-2, was increased in unchallenged *Ptpn2*^∆IEC^ mice compared to their *Ptpn2*^fl/fl^ controls. This aligns with our prior work showing that reduced PTPN2 expression or activity *in vivo* and *in vitro* results in bimodal upregulation of claudin-2 at tight junctions through transcriptional and trafficking events.^47,48^ Claudin-2 is upregulated in inflammatory states and is shown to increase paracellular electrolyte permeability via the pore pathway, while it also directly increases paracellular water flux.^49,50^ Interestingly, claudin-2 is also upregulated during infections with *C. rodentium* and it mediates diarrhea and clearance of invasive bacteria, thus reflecting a protective role against infection.^36^ Consistent with this study, we observed in *Ptpn2*^fl/fl^ mice that claudin-2 was upregulated in response to *m*AIEC infection. Interestingly, the increased background claudin-2 in *Ptpn2*^∆IEC^ mice did not mediate a predicted protective effect against infection. Indeed, claudin-2 levels were suppressed in these mice to levels comparable to uninfected *Ptpn2*^fl/fl^ mice. Thus, it appears that loss of PTPN2 in intestinal epithelium compromises multiple-host defense mechanisms including AMP production, intestinal permeability, and claudin-2 mediated paracellular water flux.

To understand the mechanisms mediating these effects, we identified that PTPN2 loss compromised local mucosal cytokine responses involved in promoting AMP production (IL-22, IL-17A) and claudin-2 (IL-6, IL-22) expression. IL-22 plays a critical role during pathogen infiltration by stimulating AMPs and preserving the mucosal barrier through epithelial regeneration.^36,51^ Furthermore, IL-22 receptors are equally expressed in *Ptpn2*^fl/fl^ and *Ptpn2*^∆IEC^ mice, therefore, we supplemented *Ptpn2*^fl/fl^ and *Ptpn2*^∆IEC^ with exogenous IL-22 to determine if the increased susceptibility and barrier dysfunction in *Ptpn2*^∆IEC^ mice could be rescued by IL-22.^52^ We observed that *Ptpn2*^fl/fl^ mice displayed a dramatic reduction of *m*AIEC bacterial load following IL-22 treatment. Furthermore, the bacterial load in *Ptpn2*^∆IEC^ was also significantly reduced. The FD4 permeability defect observed in *Ptpn2*^∆IEC^ mice post-*m*AIEC^red^ infection was also reversed after reconstitution of IL-22 but the same effect was not seen in the *Ptpn2*^fl/fl^ - *m*AIEC^red^ group. We also identified that the barrier forming proteins E-cadherin and occludin, which were lower in *Ptpn2*^∆IEC^-*m*AIEC mice, were restored after IL-22 administration. Moreover, we also demonstrated elevated expression of antimicrobial proteins (lysozyme, Defa5, Defa6) and the antimicrobial processing enzyme, MMP-7, in *Ptpn2*^∆IEC^-*m*AIEC mice after reconstitution of IL-22. These data strongly indicate that the deficiency in IL-22 production was likely crucial to the barrier defects observed in infected *Ptpn2*^∆IEC^ mice.

Our results are in line with other studies showing that IL-22 is necessary for host defense against infiltrating pathobionts.^51,53–55^ Moreover, they align with our prior observations that *Ptpn2*^∆IEC^ mice fail to increase IL-22 production following *C. rodentium* infection.^52^ We previously showed that loss of PTPN2 in macrophages elevated IL-22 production in *C. rodentium* infected *Ptpn2*LysMCre mice and macrophage IL-22 was required for the faster recovery from infection seen in these mice. Notably, *Ptpn2*^∆IEC^ mice failed to mount an IL-22 response to *Citrobacter* infection.^52^ While in the current study we did not identify which IL-22 producing cells failed to respond in *Ptpn2*^∆IEC^-*m*AIEC mice, based on our prior data with *Ptpn2*LysMCre mice,^52^ it is not unreasonable to suggest that the normal presence of PTPN2 in macrophages in *Ptpn2*^∆IEC^ mice acted as a brake on macrophage IL-22 production in response to infection. Of likely greater significance from the perspective of epithelial PTPN2 regulation of host responses to infection, is that while our current study showed that recombinant IL-22 reduced bacterial load and restored epithelial alpha-defensin expression in *Ptpn2*^∆IEC^ mice following AIEC infection, rIL-22 failed to reduce bacterial load and restore epithelial Reg3gamma antimicrobial peptide expression in *C. rodentium* infected *Ptpn2*^∆IEC^ mice.^52^ This suggests that loss of epithelial PTPN2 can provoke diverse signaling outcomes, and differential responses to cytokines, following infection with mucosal pathobiont (*m*AIEC) vs. pathogenic (*C. rodentium*) *E. coli*. While the mechanism(s) responsible for these divergent responses to different bacterial strains remains to be elucidated, one possible clue may lie in a skewing toward a dominant STAT1 phosphorylation response in PTPN2-defici ent cells. This was consistently observed in naïve and infected *Ptpn2*^∆IEC^ mice and will be explored in future studies.^52^ Our prior study also showed that mice lacking one copy of the *PTPN2* gene in all cells (whole-body *Ptpn2* heterozygous mice) also exhibited a similar phenotype to *Ptpn2*^∆IEC^ mice with respect to delayed recovery from *C. rodentium* infection and a reduced ability to respond to IL-22 expressing macrophages.^52^ Clinically, these mice most closely align with individuals carrying *PTPN2* loss-of-function variants and suggest that even a partial reduction in PTPN2 activity is sufficient to compromise mucosal immune responses. Taken together, these data demonstrate that the loss of PTPN2 in epithelial cells disturbs the gut micro-environment, rendering it more susceptible to infection or modulation by commensal bacteria with pathobiont potential.

The intestinal epithelium plays a critical yet nuanced role in maintaining homeostasis between the subepithelial immune cells and the luminal microbes. The epithelium must strike a delicate balance to prevent invading microbes but allow selective permeability necessary for nutrient and electrolyte absorption. Our findings highlight that intestinal epithelial PTPN2 is crucial for mucosal immunity as it promotes antimicrobial peptide defenses and enhances barrier function during infection from pathobionts such as AIEC, and noninvasive commensals. In addition, we identify a unique mechanism for epithelial PTPN2 in maintaining the immune-cytokine landscape of the gut post pathobiont infiltration. These findings reveal an essential role for epithelial expression of this clinically important gene in the maintenance of key defense molecules, barrier function, and the coordinated immune landscape mediating gut homeostasis.

## Supplementary Material

Supplementary Figures and Legends.pdf

supp figures and grapical abstract.zip
