# Supplementary material for: Intestinal epithelial PTPN2 limits pathobiont colonization by immune-directed antimicrobial responses
Source: Gut Microbes. 2025 Sep 15;17(1):2559029. doi: 10.1080/19490976.2025.2559029 (PMC12445515; doi:10.1080/19490976.2025.2559029)

Supplementary Figure Legends

Supplementary Figure 1. Validation of *m*AIEC^{red}

Caco-2BBe cells were infected with PBS, *E. coli* K12, *m*AIEC and *m*AIEC^{red}. (A) *m*AIEC and *m*AIEC^{red} display similar (A) Adherence (B) Invasion of Caco-2BBe human IECs. Both had higher invasion compared to *E. coli* K12 (*P*=0.0023) and PBS was taken as a negative control. (C) C57Bl/6 mice were infected with *m*AIEC^{red} at 10^{9} cfu/mL and *m*AIEC^{red} was detected in mouse fecal contents for a period of 21 days. (D) Immunofluorescence was performed on Caco-2BBe cells infected with *m*AIEC^{red}. Epithelial cells are marked with F-actin (green) and bacteria are seen in red. Images demonstrate that *m*AIEC^{red} can invade intestinal epithelial cells.

Supplementary Figure 2. *Ptpn2*$^{fl/fl}$ and *Ptpn2*$^{\Delta IEC}$ have similar luminal bacterial load

Bacterial load was enumerated from *Ptpn2*$^{fl/fl}$ and *Ptpn2*$^{\Delta IEC}$ mice. (A), (B) Similar *E. coli* burden was observed in the proximal colon, distal colon luminal contents between *Ptpn2*$^{fl/fl}$ and *Ptpn2*$^{\Delta IEC}$ mice treated with PBS and K12. (D, E) Comparable *m*AIEC^{red} colonization was observed between *Ptpn2*$^{fl/fl}$ and *Ptpn2*$^{\Delta IEC}$ *m*AIEC infected groups. Groups were compared with 2-way ANOVA with Tukey multiple comparisons test. **P*<0.05, ***P*<0.01, *P****0.001.

Supplementary Figure 3. Loss of epithelial *Ptpn2* leads to reduced expression of CD3 and CD45 protein levels.

Ptpn2$^{fl/fl}$ and *Ptpn2*$^{\Delta IEC}$ whole tissue proximal colon was processed for western blot (A), Representative western blot for CD3, CD45 protein expression. *Ptpn2*$^{\Delta IEC}$ mice display reduced (B) CD45 protein expression (C) CD3 protein expression after *m*AIEC infection compared to *Ptpn2*$^{fl/fl}$- *m*AIEC group. (D) *Ptpn2*$^{\Delta IEC}$ mice display higher p-STAT1 protein levels in PBS and infected mice. Groups were compared with 2-way ANOVA with Tukey multiple comparisons test. **P*<0.05, ***P*<0.01, *P****0.001.

Supplementary Figure 4. Loss of epithelial *Ptpn2* does not alter expression of β-defensins.

Ptpn2$^{\Delta IEC}$ and *Ptpn2*$^{fl/fl}$ whole tissue proximal colon samples were processed for mRNA quantification. *Ptpn2*$^{\Delta IEC}$ mice do not display any changes in (A) *Defb1*, (B) *Defb2* or (C) *defb3* response compared to control or infected littermates. Groups were compared with 2-way ANOVA with Tukey multiple comparisons test. **P*<0.05, ***P*<0.01, *P****0.001.

Supplementary Figure 5. Loss of epithelial *Ptpn2* does not change Th2 cytokine responses.

Ptpn2$^{\Delta IEC}$ and *Ptpn2*$^{fl/fl}$ whole tissue proximal colon samples were processed for mRNA quantification. *Ptpn2*$^{\Delta IEC}$ mice do not display any changes in (A) *Il4*, (B) *Il13* or (C) *Gata3* response compared to control or infected littermates. Groups were compared with 2-way ANOVA with Tukey multiple comparisons test. **P*<0.05, ***P*<0.01, *P****0.001.

Supplementary Figure 1

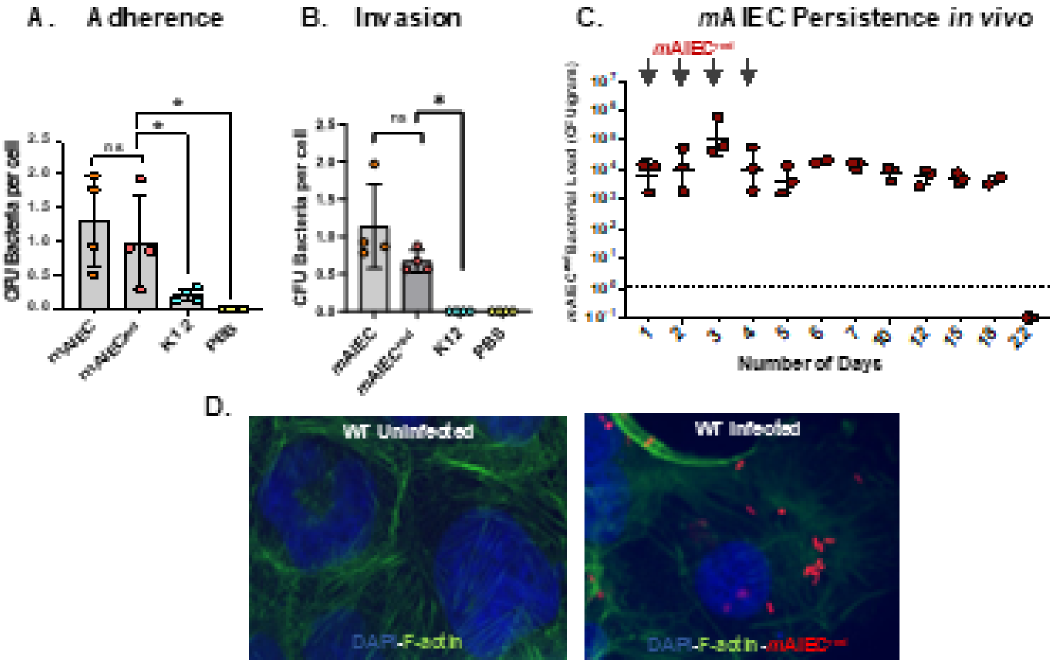

Supplementary Figure 2

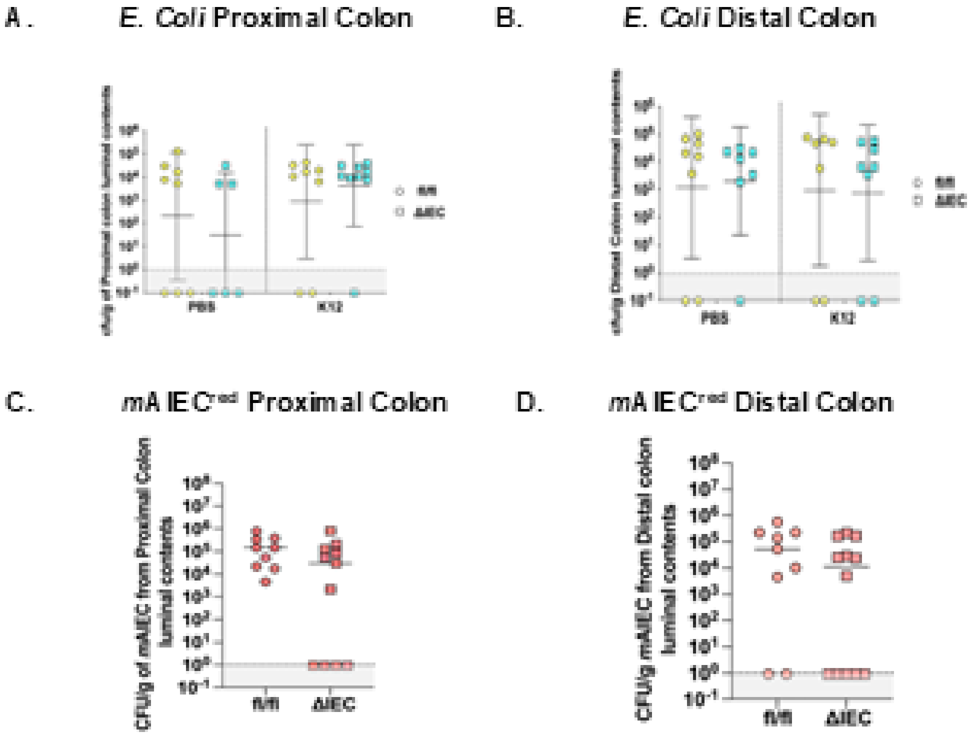

Supplementary Figure 3

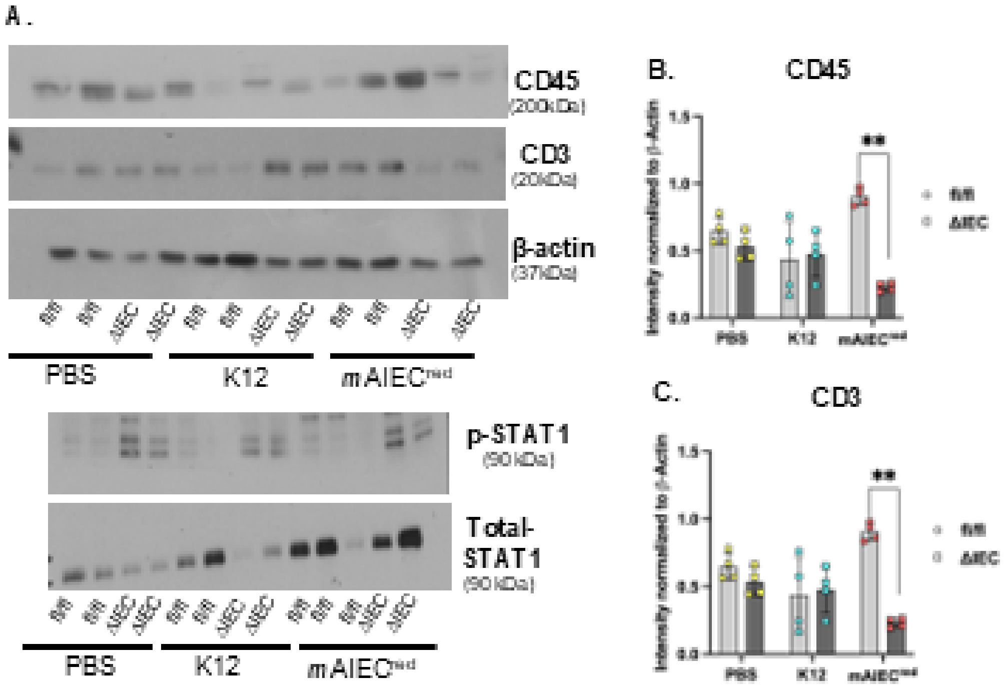

Supplementary Figure 4

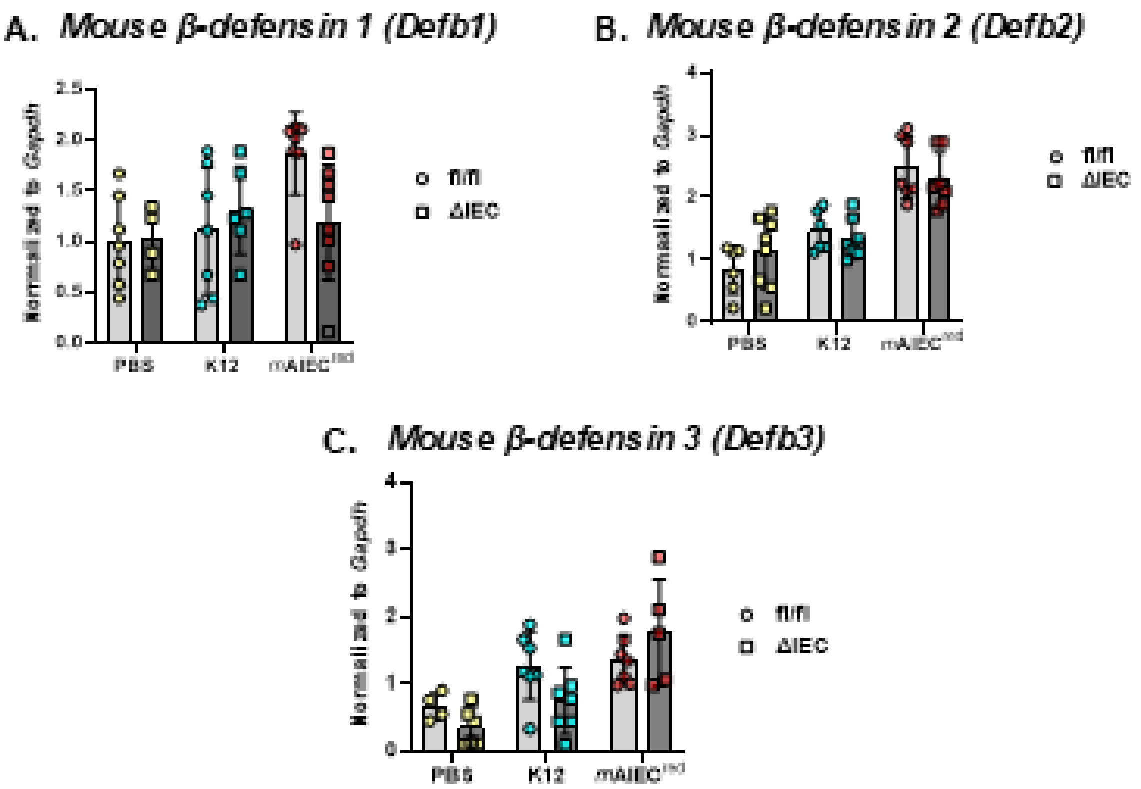

Supplementary Figure 5

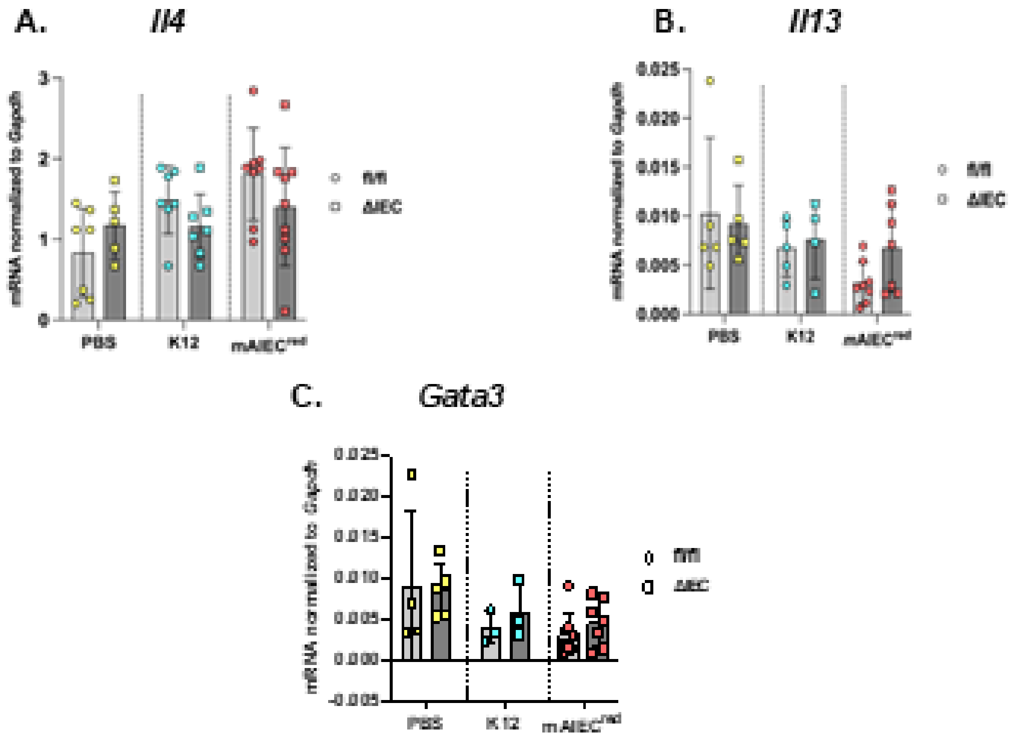

Supplement: Supplementary Figures and Legends.pdf [file KGMI_A_2559029_SM9298.pdf]
