# Supplementary figures and images for: Intestinal epithelial PTPN2 limits pathobiont colonization by immune-directed antimicrobial responses
Source: Gut Microbes. 2025 Sep 15;17(1):2559029. doi: 10.1080/19490976.2025.2559029 (PMC12445515; doi:10.1080/19490976.2025.2559029)

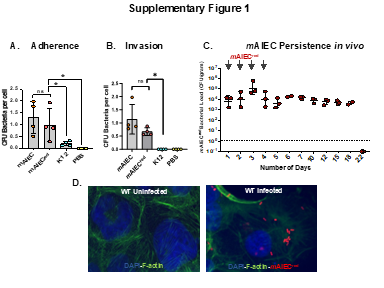

Supplement: supp figures and grapical abstract.zip [file KGMI_A_2559029_SM9297.zip › Figure_S1.tif]

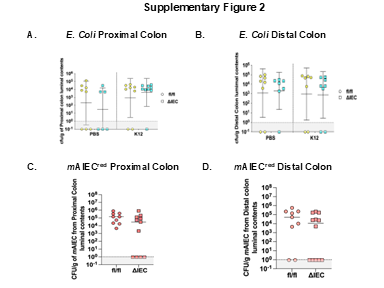

Supplement: supp figures and grapical abstract.zip [file KGMI_A_2559029_SM9297.zip › Figure_S2.TIF]

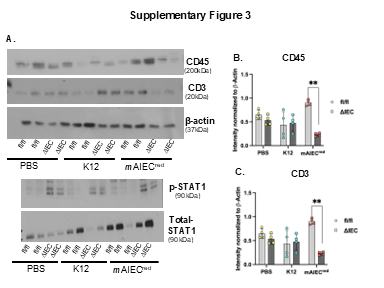

Supplement: supp figures and grapical abstract.zip [file KGMI_A_2559029_SM9297.zip › Figure_S3.TIF]

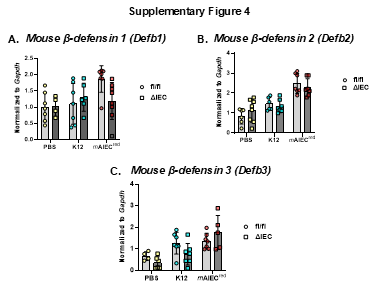

Supplement: supp figures and grapical abstract.zip [file KGMI_A_2559029_SM9297.zip › Figure_S4.TIF]

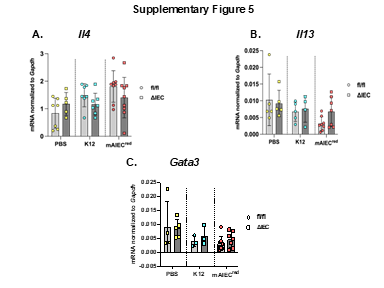

Supplement: supp figures and grapical abstract.zip [file KGMI_A_2559029_SM9297.zip › Figure_S5.TIF]

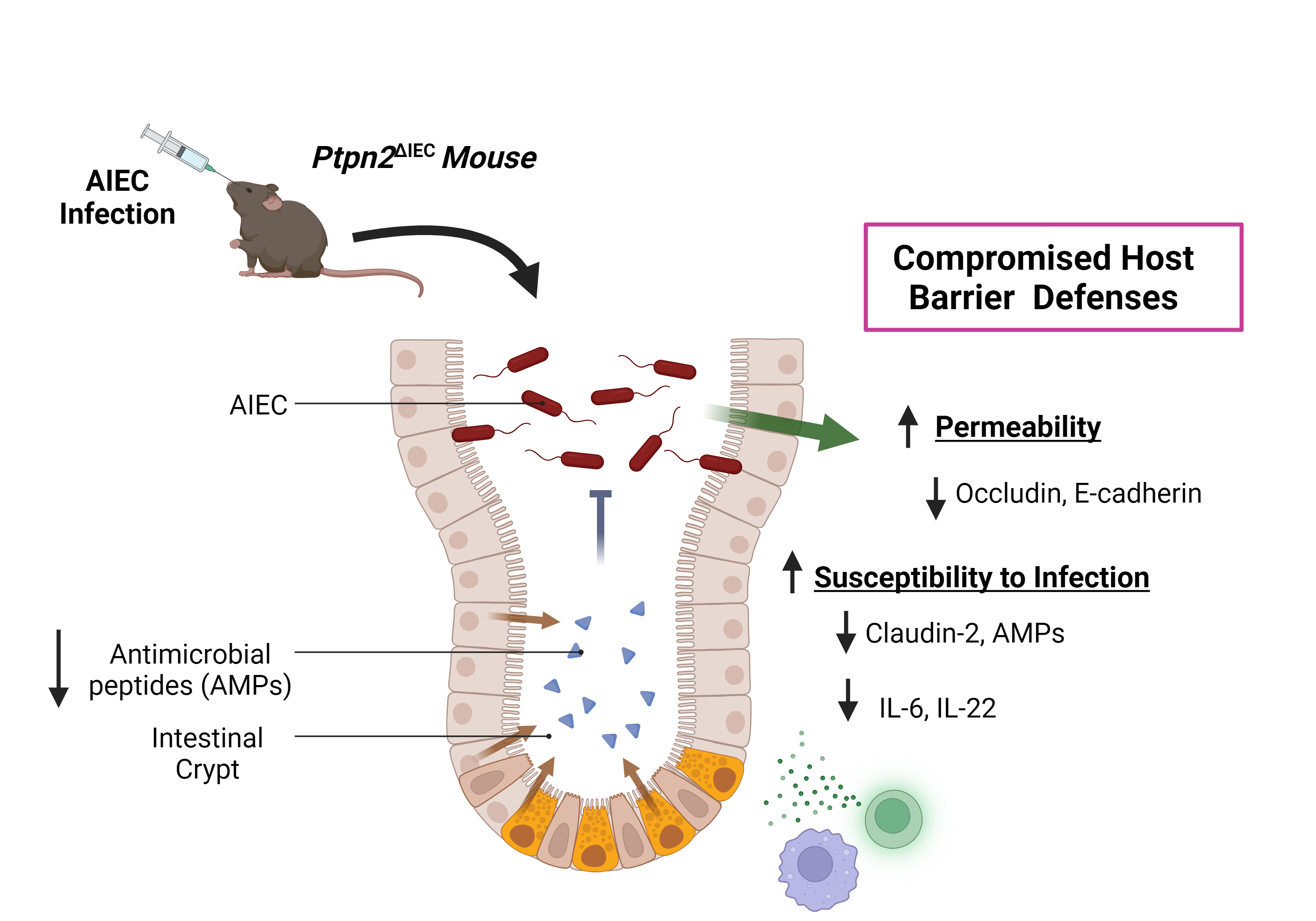

Supplement: supp figures and grapical abstract.zip [file KGMI_A_2559029_SM9297.zip › Graphical_Abstract_mAIEC_Barrier_Manuscript.png]
